# Design, Conduct, and Use of Patient Preference Studies in the Medical Product Life Cycle: A Multi-Method Study

**DOI:** 10.3389/fphar.2019.01395

**Published:** 2019-12-03

**Authors:** Eline van Overbeeke, Rosanne Janssens, Chiara Whichello, Karin Schölin Bywall, Jenny Sharpe, Nikoletta Nikolenko, Berkeley S. Phillips, Paolo Guiddi, Gabriella Pravettoni, Laura Vergani, Giulia Marton, Irina Cleemput, Steven Simoens, Jürgen Kübler, Juhaeri Juhaeri, Bennett Levitan, Esther W. de Bekker-Grob, Jorien Veldwijk, Isabelle Huys

**Affiliations:** ^1^Clinical Pharmacology and Pharmacotherapy, KU Leuven, Leuven, Belgium; ^2^School of Health Policy & Management, Erasmus University Rotterdam, Rotterdam, Netherlands; ^3^Centre for Research Ethics and Bioethics, Uppsala University, Uppsala, Sweden; ^4^Muscular Dystrophy UK, London, United Kingdom; ^5^John Walton Muscular Dystrophy Research Centre, Newcastle University, Newcastle, United Kingdom; ^6^Pfizer, Tadworth, United Kingdom; ^7^Applied Research Division for Cognitive and Psychological Science, European Institute of Oncology, Milan, Italy; ^8^Department of Oncology and Hematology Oncology, Faculty of Medicine and Surgery, University of Milan, Milan, Italy; ^9^Belgian Health Care Knowledge Centre, Brussels, Belgium; ^10^Quantitative Scientific Consulting, Marburg, Germany; ^11^Sanofi, Bridgewater, NJ, United States; ^12^Janssen Research & Development, Titusville, NJ, United States

**Keywords:** patient preferences, medical products, decision-making, health technology assessment, marketing authorization

## Abstract

**Objectives:** To investigate stakeholder perspectives on how patient preference studies (PPS) should be designed and conducted to allow for inclusion of patient preferences in decision-making along the medical product life cycle (MPLC), and how patient preferences can be used in such decision-making.

**Methods:** Two literature reviews and semi-structured interviews (n = 143) with healthcare stakeholders in Europe and the US were conducted; results of these informed the design of focus group guides. Eight focus groups were conducted with European patients, industry representatives and regulators, and with US regulators and European/Canadian health technology assessment (HTA) representatives. Focus groups were analyzed thematically using NVivo.

**Results:** Stakeholder perspectives on how PPS should be designed and conducted were as follows: 1) study design should be informed by the research questions and patient population; 2) preferred treatment attributes and levels, as well as trade-offs among attributes and levels should be investigated; 3) the patient sample and method should match the MPLC phase; 4) different stakeholders should collaborate; and 5) results from PPS should be shared with relevant stakeholders. The value of patient preferences in decision-making was found to increase with the level of patient preference sensitivity of decisions on medical products. Stakeholders mentioned that patient preferences are hardly used in current decision-making. Potential applications for patient preferences across industry, regulatory and HTA processes were identified. Four applications seemed most promising for systematic integration of patient preferences: 1) benefit-risk assessment by industry and regulators at the marketing-authorization phase; 2) assessment of major contribution to patient care by European regulators; 3) cost-effectiveness analysis; and 4) multi criteria decision analysis in HTA.

**Conclusions:** The value of patient preferences for decision-making depends on the level of collaboration across stakeholders; the match between the research question, MPLC phase, sample, and preference method used in PPS; and the sensitivity of the decision regarding a medical product to patient preferences. Promising applications for patient preferences should be further explored with stakeholders to optimize their inclusion in decision-making.

## Highlights

 - Collaboration among stakeholders in patient preference studies (PPS) improves value and acceptance;
 - The patient sample and method should match the medical product life cycle (MPLC) phase;
 - The preference method should be matched to the research question and patient population;
 - Patient preference sensitivity of decisions regarding medical products impacts the value of PPS;
 - Applications have to be further explored to allow for systematic use of PPS results in MPLC decision-making.

## Introduction

Patient preference information is defined by the United States, (US) Food and Drug Administration (FDA) as “qualitative or quantitative assessments of the relative desirability or acceptability to patients of specified alternatives or choices among outcomes or other attributes that differ among alternative health interventions” (FDA, 2016). Patient preference information, or patient preferences, reflects what treatment attributes matter to patients, how much these matter to patients and how patients make trade-offs between treatment attributes. Patient preferences can be estimated through the conduct of patient preference studies (PPS) using preference exploration (qualitative) or elicitation (quantitative) methods. Preference exploration methods can be defined as “*qualitative methods that collect descriptive data through participant or phenomenon observation, and examine the subjective experiences and decisions made by participants*” (Soekhai et al., 2019). Examples of preference exploration methods include semi-structured interviews and focus groups. Preference elicitation methods can be defined as “*quantitative methods collecting quantifiable data that can be reported through statistical inferences or analysis*” (Soekhai et al., 2019). Examples of preference elicitation methods include discrete choice experiments (DCE), analytical hierarchy process (AHP), and standard gamble. While health preference research has existed for over 40 years, the use of PPS in decision-making along the lifecycles of drugs and medical devices (called the medical product lifecycle; MPLC) is limited but gaining attention (Craig et al., 2017).

As with the adoption of all new concepts according to Roger's Diffusion of Innovation Theory (Barrow and Toney-Butler, 2019), the implementation of patient preferences in decision-making depends on stakeholders' understanding and acceptance of patient preferences as well as how this new concept can be put into practice. Important stakeholders in this context are patients as they are the ones potentially participating in PPS and the ones affected by the decisions that can be informed with the study results. The pharmaceutical and medical device industry is another important stakeholder as they are the potential sponsors of PPS; additionally, PPS's results may alter the course of development of their products. Last but not least, regulators and HTA bodies and payers will be confronted with the findings of PPS during their assessments.

Stakeholders seem to agree on the potential value of patient preferences in decision-making along the MPLC (de Bekker-Grob et al., 2017). Patient preferences are found to provide additional information on medical products, such as insights into the relative importance to patients of clinical outcomes and safety issues. Moreover, they can lead to more relevant, well-informed, transparent, publically trusted, and patient-centric decisions (van Overbeeke et al., 2019). While there seems to be agreement on the value of PPS, there is a lack of guidance on how to conduct PPS aiming to inform decision-making and on how patient preferences can be used in assessments and decision-making, possibly explaining the limited use of these studies (van Overbeeke et al., 2019).

The aim of this study was to investigate stakeholder perspectives on how PPS should be designed and conducted to allow for inclusion of patient preferences in decision-making along the MPLC; moreover, this study investigates how patient preferences can be used in such decision-making.

## Methods

### Multi-Method Design

**Figure 1:**
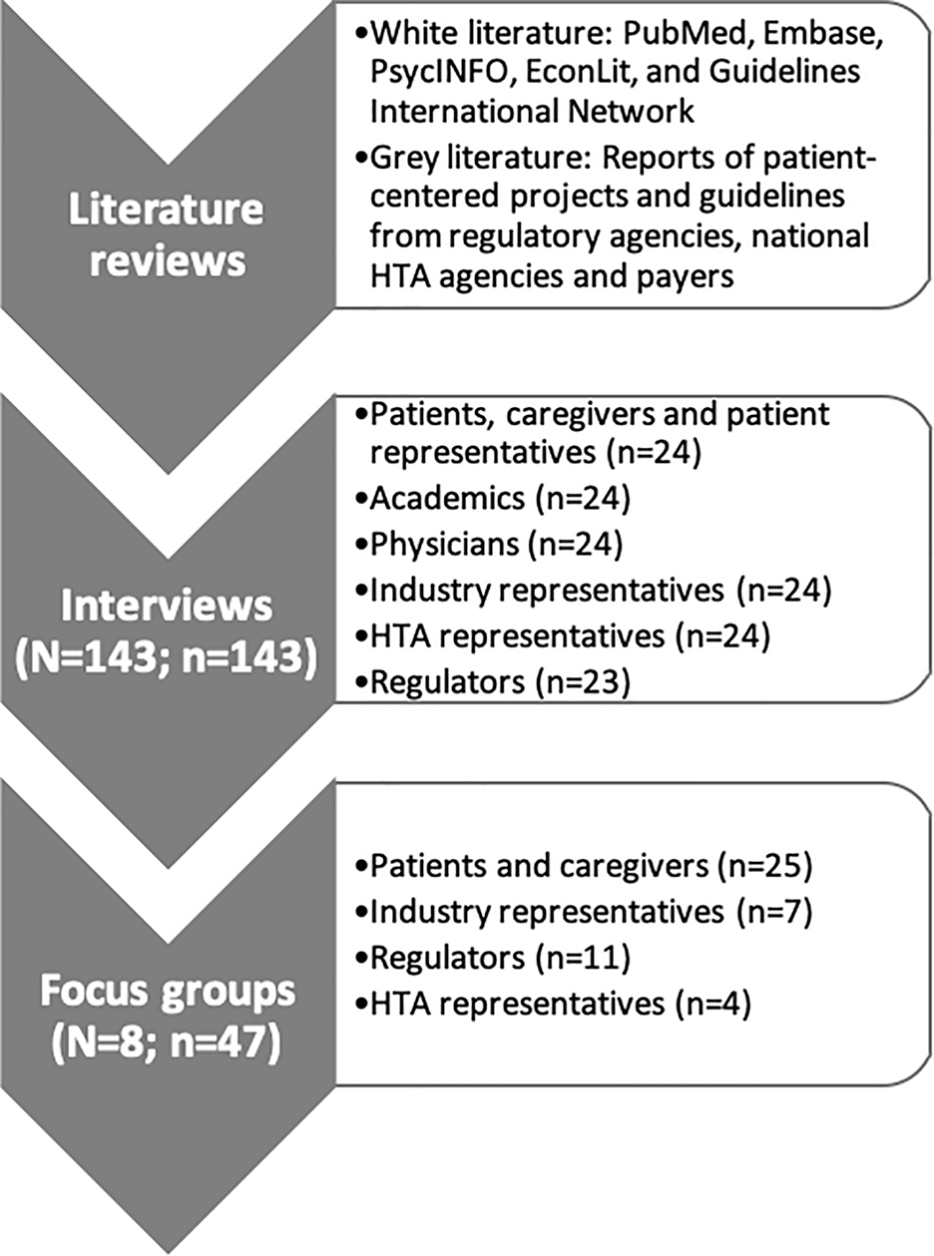
Methods used in the multi-method design. First, two literature reviews were performed to identify 1) factors and situations that influence the value of patient preference studies (PPS) in decision-making along the MPLC (van Overbeeke et al., 2019), and 2) the potential roles, expectations, concerns and requirements associated with using patient preferences (Janssens et al., 2019). Second, interviews (n = 143) with different healthcare stakeholders were held to address the same research questions (Whichello et al., 2019; Janssens et al., 2019). Lastly, focus groups (n = 8) were conducted to discuss topics related to the design, conduct and use of PPS for which opinions of interviewees differed or deeper understanding was needed. Abbreviations: HTA, Health Technology Assessment; N, number of interviews/focus groups; n, number of participants.

This study was conducted in the context of the “Patient Preferences in Benefit-Risk Assessments during the Drug Life Cycle” (PREFER) project. The PREFER project aims “*to strengthen patient-centric decision-making throughout the life cycle of medicinal treatments by developing expert and evidence-based recommendations on how patient preferences should be assessed and inform decision-making*” (de Bekker-Grob et al., 2017). Two literature reviews and 143 interviews with healthcare stakeholders in Europe and the US were conducted (**Figure 1**) to identify stakeholders' needs, factors influencing the value of PPS for decision-making, and potential applications of patient preferences along the MPLC. Methods and results of literature reviews have been published elsewhere (Janssens et al., 2019; van Overbeeke et al., 2019), as well as those of the interviews (Janssens et al., 2019; Whichello et al., 2019). Several topics remained unanswered and group discussions were deemed necessary to provide more insight and to give further direction on the potential implementation of patient preferences in the MPLC.

### Design of Focus Group Guides

Focus group guides (**Supplementary Material I**) were designed with patients, industry representatives, regulators and HTA representatives to include topics on which opinions differed during the interview phase or deeper understanding was needed. Selected *patient* focus group topics were: important treatment attributes, preferred designs of PPS, facilitators and barriers for participating in PPS, and data management. Selected topics for *industry, regulatory and HTA* focus groups included: representativeness of the patient sample, use of patient preferences, roles of different parties involved in PPS and data management in PPS. Before these topics were discussed, the definition of patient preferences and an example of a PPS were debated with participants.

### Participant Recruitment

A total of eight focus groups of 3–10 participants each were conducted (**Figure 1**). Four patient focus groups were conducted in Europe, across four disease areas that vary in prevalence, level of unmet need, chronicity, and cause (**Table 1**). In addition, four stakeholder-specific focus groups were held with European industry representatives, European regulators, US regulators, and European and Canadian HTA representatives, respectively. Participants were recruited *via* patient organizations, hospitals, the European Federation of Pharmaceutical Industries and Associations (EFPIA), the European Medicines Agency (EMA), the FDA, and the HTA advisory group of PREFER. Participants were selected through purposive sampling, on the basis of their experiences and expertise. Focus groups with regulators specifically included experts on drugs, biologics, advanced therapies, orphan drugs, medical devices, and patient engagement. In the industry focus group, participants were included from research and development, regulatory affairs, market access, and patient engagement departments.

**Table 1:** Patient and caregiver demographics, clinical information and health literacy

Characteristics	Patients and caregivers (n = 25)	
	n	%
Sex		
Females	10	40
Males	15	60
Age, years		
18-24	0	0
25-39	1	4
40-60	9	36
> 60	15	60
Country		
United Kingdom	4	16
Italy	8	32
Romania	10	40
Sweden	3	12
Education		
No diploma	4	16
High school	11	44
Bachelor's degree	9	36
Master's degree	0	0
PhD	1	4
Stakeholder group		
Patient	22	88
Caregiver	2	8
Patient and caregiver	1	4
Disease area		
Neuromuscular disorders	4	16
Lung cancer	8	32
Cardiovascular diseases	10	40
Rheumatoid arthritis	3	12
Years since diagnosis		
< 1	2	8
1-3	8	32
4-10	5	20
> 10	10	40
Health literacy		
Adequate health literacy	20	80
Inadequate health literacy	5	20

### Conduct

Patient focus groups were conducted face-to-face in the native language of the patients and others - industry, regulatory and HTA focus groups – *via* teleconferences in English, between September 2017 and April 2018. Patients were asked to fill in a demographics survey, including questions on their disease and treatment history, as well as health literacy (**Table 1**). Health literacy was assessed using the three health literacy screening questions of Chew et al. (2008), an instrument “*based on an individual's level of self-reported difficulty with understanding information or performing reading tasks they encounter in the health care setting*” (Chew et al., 2008). Focus groups lasted between 45 min and 2 h and were recorded, transcribed verbatim, and pseudonymized. Non-English transcripts were translated to English.

### Analysis

Demographic characteristics were analyzed using descriptive statistics. Health literacy scores were calculated (Chew et al., 2008; Fransen et al., 2011). Thematic analysis of the focus group transcripts, as described by Howitt (Howitt, 2016), was performed by two researchers (EO and RJ) to minimize variability of interpretation (full analysis plan available in **Supplementary Material II**). The data were investigated for patterns, consensuses, and critical observations across focus groups, which resulted in the creation of thematic 'codes'. First, the researchers familiarized themselves with the content of the focus groups by moderating or assisting the focus groups and reading the transcripts. Familiarization resulted in the establishment of an overview of the collected data, and the researchers became aware of key themes and concepts. Subsequently, the researchers independently identified topics throughout the transcripts and afterwards agreed upon a list of initial themes. The researchers reread the transcripts of the focus groups and formulated a list of final themes. The text of the transcripts was organized by applying the themes using NVivo qualitative data analysis software by QRS International Pty Ltd Version 12, 2018. Sections of text that corresponded to a theme were indexed, and placed in charts with headings reflecting these themes. These charts were then analyzed and interpreted for common attitudes and opinions of the participants, with comparisons being made between focus groups. Definitions were given to themes (**Supplementary Material III**) and the results were described.

## Results

Results were analyzed per stakeholder group and subsequently grouped per theme. Codes following quotations refer to participants' characteristics: CA, caregiver; CAN, Canada; CVD, cardiovascular disease; EU, Europe; HTA, health technology assessment representative; IN, industry representative; LC, lung cancer; DM, myotonic dystrophy; PA, patient; RA, rheumatoid arthritis; RE, regulator; US, United States.

### Design and Conduct of Patient Preference Studies

#### Research Questions

For PPS to be valuable for decision-making, industry and regulatory representatives mentioned that such studies should investigate research questions related to trade-offs between benefits and risks, and between benefits and administration attributes. Administration attributes of interest included drug formulation, administration frequency, time investment and place of administration (e.g. home vs. hospital). These trade-offs were found to be informative of the risks and administration inconveniences patients would tolerate. HTA representatives, and also regulators, were interested in what clinical endpoints and other attributes patients prefer. HTA representatives stated that if they would know what outcomes patients prefer, they could assess whether treatments fulfill these needs. In addition, regulators would like PPS to provide insights on the burden of disease and were interested in methodological research questions related to PPS, e.g. how results from different methods compare in the same population and how results from different samples differ, including samples from different countries.

#### Representativeness of Participants

All stakeholders were asked who should participate in PPS. CVD, and RA patients agreed that patients with different disease and treatment experiences should participate and that patients should not be excluded based on age, education or profession. CVD and DM participants and HTA representatives suggested to explore caregiver preferences instead of patient preferences in some specific cases: when patients have impaired cognitive function or in case patients cannot be asked about their preferences. Moreover, MD participants mentioned that including severely affected MD patients in a PPS might be problematic in cases where MD patients do not accept their illness and asking about their preferences would give a wrong impression: “*because you have put them under pressure* … *you make them uncomfortable so you have not got the right response*” (CA1_UK_MD). HTA representatives emphasized that if caregiver preferences are submitted, it should be justified why patient preferences were not explored. One RA and one DM patient also suggested that the patient sample should be representative of the patient population.

Industry and regulatory representatives mentioned that the representativeness requirements for the sample depend on the MPLC phase, the methods used, and the claimed indication. While for earlier MPLC phases industry representatives advocated for a very diverse sample including different countries and cultures, in later phases samples should be representative of the international or national population for which a decision needs to be made. Moreover, according to industry and regulatory representatives, in qualitative studies representativeness would be less important than in quantitative studies. In industry, regulatory and HTA focus groups it was agreed that quantitative PPS samples should be as representative as possible of the patient population to allow for generalization of results. Non-representativeness was stated to lead to non-acceptance of the data by regulators. The meaning of a representative sample was further discussed; industry, regulatory and HTA representatives mentioned that the sample should be representative of the population that is targeted with the therapy, including those that would refuse treatment. In the context of rare diseases, EU regulators expressed that it could not be expected to reach the level of representativeness as in common diseases but that agreements should be made on the approach of selecting the patient sample.

To ensure representativeness of the patient sample, regulators found that a structured research question and disease area specific approach to define the patient sample should be set-up early in the design phase of the study. Moreover, regulators suggested to do sample size calculations and together with industry and HTA representatives stated that available epidemiological data should be investigated to assess population heterogeneity and understand preference heterogeneity when PPS results are available. It was recognized that reaching representativeness is a difficult exercise and that it is “*a balance between methodological rigor and pragmatism*” (HTA2_EU). Diversity and representativeness should be ensured through a set of criteria, stratified sampling or other sampling schemes. According to industry and regulatory representatives, the patient sample should ideally cover different age groups, genders, cultures, ethnicities, geographical areas (continents, countries or regions depending on what decisions are aimed to inform), levels of education, time since diagnosis, stages and severities of disease, and treatment experiences. Also, different recruitment channels should be explored to reach the different types of patients (e.g. patient organizations and physicians). While industry representatives agreed on the previous requirements, they had differing opinions on whether “*average*” patients or expert patients should be included in the sample.

#### Method and Instrument Design

Method and instrument design of PPS was discussed in all focus groups, including the kind of information patients should receive when participating in PPS, instrument and question design, question administration, and factors influencing participation. Patients and caregivers had different preferences for surveys, interviews, and focus groups as methods to estimate preferences. Some LC and RA patients advocated the use of surveys because “*one can take the time to think about it*” (PA4_IT_LC). Patients suggested that surveys should not be too long, involve little writing, and allow for completion from home at a time of their choice. Across LC and CVD patients, opinions differed on whether surveys should be administered online at home or at the hospital. LC patients that preferred interviews, stated that this technique allows for a more personal approach. Both RA and DM patients and caregivers were positive about the idea of group discussions, as it would allow them to discuss things “*outside the framework*” (PA1_SE_RA) and “*because people say something and then they trigger something in yourself*” (CA1_UK_DM). DM participants underlined the importance of organizing this discussion among a small number of participants, as seeing people with the same suffering can be confronting. Local meetings for DM patients and their caregivers ('support groups') would be the most optimal place for this disease group to have focus groups. As further detailed in **Supplementary Material V**, other facilitators and barriers to participate in PPS related to the relevance and possible impact of the research, hope, encouragement of caregivers, the relationship with the recruiting person, self-perception and disease acceptance, interaction with other patients, financial compensation, convenience, attractiveness and feedback on results. Discussions with patients and caregivers also revealed general categories of attributes to be considered in the design of these studies, namely benefits, risks, price, quality, administration, packaging, and storage. LC and RA patients explained their information needs during preference studies; these needs included purpose and topic of the study, ethical review, treatment-related information, and more specifically, information on attributes, including the side effects, expected therapeutic effects, and dosage form.

Question design and patient education during PPS was discussed. While one industry representative stated “*There's no reason why you can't educate a patient to understand the questions that are being asked*” (IN4_EU), all regulators and HTA representatives as well as the other industry members found it important that questions are simple and questions are adapted to the patients, not the patient sample to the method. They believed that if the method comes with a cognitive burden that is too high for the patients, the method should be changed; moreover, patients should not be excluded because they are not able to understand the questions. Industry, regulatory, and HTA representatives expressed the need to provide patients with information on the study and sufficient unbiased information on the questions to enable patients to make choices. Overall, industry representatives underlined the importance of rigor, validity, and robustness of study design to increase acceptance and use of results by regulators, HTA bodies and payers. Regulators accentuated the importance of performing qualitative studies prior to quantitative studies; “*good qualitative research is going to be incredibly important in order to get to any good design of quantitative preference study*” (RE2_EU). Moreover, industry discussed that in early stages of development qualitative methods might be more appropriate to get some initial insights and to see if the product could fulfill any high unmet needs, and that quantitative methods could provide scientific preference evidence in later stages.

#### Organizational Considerations

Organizational considerations for PPS were discussed, including when to conduct these studies, financial considerations, roles of stakeholders in design and conduct, and data management of preference data. According to some industry representatives, PPS should be planned early in the MPLC and included in the development plan to be conducted during pre-clinical or clinical phases. Some were concerned that the conduct of PPS would extend the duration of the development phase. HTA representatives believed these studies to be costly and long in duration.

The main organizational discussion point was the roles of stakeholders in design and conduct of PPS. According to industry, expert patients should be involved to ensure that questions are understandable, and regulators should be involved to ensure a rigorous design acceptable to regulators. HTA representatives stated clearly that they could not fund and conduct PPS. Both HTA and regulators expected industry to fund and conduct PPS in collaboration with independent parties like academics, physicians, patients and patient organizations, to overcome possible biases from industry, in “*public-private partnerships*” (HTA4_CAN) or with multiple companies that “*could challenge each other*” (HTA4_CAN). Regulators and HTA bodies could also be involved, but HTA representatives mentioned that this is challenging for them because of conflict of interest and because it is “*out of their comfort zone*” (HTA4_CAN). The best way to involve them would be through scientific advice. US regulators stated that, if the design of the study is appropriate, it does not matter who conducts the study.

On ownership of patient preference data, HTA representatives argued that these data should be kept in the public domain and should not be owned by a single company since patient preferences are not specific for one product; this also in order to avoid duplication of efforts and research fatigue within patient populations. Patients and caregivers believed that hospitals might prove trustable data storage sites. Differing opinions were expressed on whether ministries of health and national insurance houses should be responsible for managing PPS data. Patients underlined the importance of treating their information in a confidential way through anonymization. Importantly however, some participants spoke about the balance between confidentiality and “*moving things forward*” (PA1_UK_DM); confidentiality should not inhibit the possibility of feeding back the results to patients nor should it stand in the way of the development of medical products that might ultimately benefit them. Participants from all disease areas agreed it would be beneficial to share information coming forth from PPS among relevant stakeholders.

### Use of Patient Preferences

#### Value of Patient Preferences

Overall, industry, regulators and HTA representatives found patient preferences valuable and important. An EU regulator stated “*At the end of the day, any patient preferences are going to be valuable at any kind of regulatory assessment, whether this is pre-, during or even doing post-authorization studies*” (RE2_EU). Some HTA representatives mentioned that current value measures like Quality Adjusted Life Years (QALYs) and Quality of Life measures did not cover the patient perspective sufficiently and additional measures were sometimes necessary. Across industry, regulators and HTA representatives it was expressed that currently the patient voice is mostly sought through direct participation of patients in discussions or though qualitative methods, and that quantitative PPS are not often submitted to regulators and HTA bodies. EU regulators and HTA representatives emphasized the importance of assessing the added value of PPS per situation. They recognized that assessing this value can pose a challenge: “*it will probably be difficult to get clear guidance where you really need to do those studies, where you have to provide data in advance*” (RE4_EU). Regulatory, industry and HTA representatives further discussed situations in which patient preferences would be of added value (**Box 1**). Overall, situations were found sensitive to patient preferences in certain cases of uncertainty in the benefit-risk profile, if assessors are not familiar with a new type of treatment or disease area, or when “*some people like some benefits, some people like other benefits*” (RE3_US).

Box 1 **Patient preference sensitive situations.**

**Table:** 

***Situation***	***Reason why patient preferences are valuable***	***Source***
Special disease areas and patient populations		
*Rare diseases, areas of unmet medical need and new disease areas*	Because limited information available and limited expertise of doctors; “*we don't quite know what we're doing*” (IN7_EU).	IN, RE
*Pediatric populations*	Because of challenges in clinical trials and disease management related to formulation and route of administration.	IN, RE
*Chronic diseases and oncology*	Because of the time patients have to live with the disease. Although some found it as important in acute diseases, and did not think it would be valuable in well-known and well-studied common diseases.	IN
*Diseases with a range of disease stages and manifestations*	Because the disease heterogeneity and differences in treatment effects can cause split views between regulators as they may not be thinking of the same types of patients.	RE
*Suspected preference heterogeneity and subgroups*	Because patient preference studies could reveal subgroups for which the treatment is of different value, although regulators also mentioned this could complicate assessments.	RE, HTA
Special side and therapeutic effects		
*Special side effects*	Because of unexpected side effects or safety issues, e.g. very toxic oncology treatments.	IN, RE
*Novel benefits*	Because it is uncertain what patients think about these novel benefits.	RE
*Symptom relief*	Because symptom relief is something that can be perceived in a different way among patients, especially if drugs have a delayed impact on symptoms.	IN
*Prolongation of life*	Because life prolongation of two or three months “*might be considered by HTA bodies or payers as not so impressive*” (IN3_EU), while patients can perceive this in a different way and may need to trade-off quality versus prolongation of life.	IN
*Minor modifications in quality of life*	Because it is difficult for evaluators to understand the impact of these minor modifications.	HTA
Special types of treatments		
*Novel therapies*	Because it is uncertain what patients think about these novel technologies like precision medicine where it could help identify “*the right patient*”; “*if you look at twins, identical twins. They both have the same disease. They are both offered the same treatment. The treatment, essentially, is going to react the same way with both of them but because of their different preferences one may choose it, one may not*” (RE1_US).	RE
*Treatments that preclude other treatments*	Because the treatment would preclude the patient from getting alternative treatments in the future.	RE
Availability of other treatments	In some disease areas where a lot of treatments are available patient preferences were found to be not very valuable by regulators. In contrast, HTA representatives found patient preferences important in cases where alternatives are available that has different effects.	RE, HTA
Regulatory requirements	Because patient preferences could become more important than those of regulators when regulatory requirements are “*old fashioned or even limited*” (IN3_EU) due to unfamiliarity with the topic. However, in the case of strict regulatory pathways or “*routine*” (IN7_EU) clinical studies and disease areas there is no added value of patient preferences.	IN
Borderline benefit-risk profiles	Because regulators may not reach consensus on a decision due to presence of uncertainties with regards to clinical endpoints.	IN, RE
Borderline cost-effectiveness ratios	Because the result of the assessment is unclear.	HTA

HTA, health technology assessment focus group; IN, industry focus group; RE, regulatory focus group.

#### Position of Patient Preferences in Industry Decision-Making

A cultural barrier toward the systematic use of patient preferences was identified by industry representatives. The cultural barrier was said to be caused by people in the industry not wanting patients to influence what they think they know best, or thinking these studies are not credible or robust enough compared to “*proper science*” (IN7_EU). In addition, industry representatives were not sure whether all authorities accept patient preference information. Industry representatives agreed on the importance of having a “*process*” (IN7_EU), “*structure*” (IN3_EU), “*framework*” (IN1_EU, IN3_EU) or “*good practices*” (IN1_EU) for measuring and using patient preferences, as these would lead to more robust results and easier integration. Industry representatives saw potential for systematic inclusion of patient preferences in benefit-risk assessments as there is a “*new requirement under the ICH guideline about including patient preference in our overall clinical overview*” (IN1_EU); referring to the 'Benefits and Risks Conclusions' section of the ICH M4E(R2) guidance (ICH, 2016). Industry, regulatory and HTA representatives further discussed the different potential applications of patient preferences along industry processes (**Box 2**).

Box 2 **Potential applications of patient preferences along industry processes.**

**Table:** 

***Application***	***Value of patient preferences***	***Source***
Disease familiarization	To know what the disease means to the patient.	IN
Medical need assessment	To understand what patients value, need, want and don't want regarding treatment options. To provide information on “*what problem they would like us to fix*” (IN7_EU) and the importance of outcomes to patients, impact on symptoms and survival.	IN
Understanding alternative treatments	To assess what treatment (medicinal products, surgery and devices) options exist for patients.	RE
Go, no-go decisions	To inform “*go, no-go type decisions made by companies*” in early development (HTA4_CAN).	HTA
Scientific advice	To have patients, or a mix of patients and patient experts, involved in scientific advice discussions.	IN
Clinical trial design	To inform clinical trial design, especially in young pediatric populations, to increase recruitment and retention of patients.	IN
*Endpoints and patient reported outcomes identification*	“*To help determine whether the outcomes are important to patients" or even "to get a better understanding of a super outcome*” (RE6_US).	IN, RE
*Designing patient information and consent forms*	To ameliorate presentation and content of patient information and consent forms.	IN
Formulation and dosage choice	To inform formulation and dosage choice, especially when there is a choice between formulations with different efficacies.	IN
Benefit-risk assessment	To assess if the delivered benefit is meaningful to patients.	IN
*Subgroup identification*	To inform regulators of subgroups where the relevance of the benefit could be more important and to show “*one size doesn't fit all*” (IN6_EU).	IN
Submission to regulators, HTA bodies and payers	To include in submissions to support the evidence and value proposition in the dossier and to allow for discussion.	IN
Labeling	To include patient preferences in labeling, reflecting what is important to patients and preventing to put black box warnings and taking medicines off the market too quickly.	IN
Product profile validation	To assess in the post-marketing phase if the product fulfils the target product profile according to patients' needs, and to assess factors influencing adherence.	RE
Informing new product development	To inform development of new products, especially for medical devices where development is “*a lot faster*” (RE6_US).	RE

HTA, health technology assessment focus group; IN, industry focus group; RE, regulatory focus group.

#### Position of Patient Preferences in Regulatory Decision-Making

According to EU regulators, PPS are not often submitted to regulatory agencies in the EU and they are not systematically considered in decision-making regarding marketing authorizations. EU regulators stated that these studies could be requested and considered on a case-by-case basis. In contrast to EU regulators, US regulators named multiple examples of decision-making where patient preferences were taken into account and explained that patient preferences are increasingly becoming an important factor within benefit-risk assessment, mainly for medical devices. While EU and US regulators and industry representatives discussed different potential applications of patient preferences along regulatory processes (**Box 3**), multiple EU regulators believed benefit-risk assessment to be the only process through which patient preferences can have an influence on the final decision; “*Quite frankly, I think it can be only in the benefit-risk. That is where the regulators will make the decision*” (RE4_EU). Within benefit-risk assessment, subgroup identification was found to be important as this would allow identification of subpopulations more willing than the majority to accept risks in order to receive certain benefits. Regulators stated that if it is demonstrated that a subpopulation would tolerate the risks associated with a certain product, that they would be willing to approve that product for the whole patient population, so that the subpopulation has the chance of receiving the treatment. Other patients would still be able to refuse the treatment in this case. Another important application mentioned by EU regulators was the assessment of major contribution to patient care. To be granted an orphan designation in Europe, developers may have to show that the product brings significant benefit, meaning a clinically relevant advantage or a major contribution to patient care compared with existing methods to treat the condition. Major contribution to patient care can include convenient modes of administration improving patient compliance, improved availability, or other arguments that may improve quality of life (Committee for Orphan Medicinal Products (COMP), 2010; European Medicines Agency (EMA), 2018). EU regulators mentioned they might request to perform PPS if major contribution to patient care is claimed. In the context of orphan drugs, an EU regulator stated “*patient preference may be the main information that we are looking at for making our regulatory decisions*”.

Box 3 **Potential applications of patient preferences along regulatory processes.**

**Table:** 

*Application*	*Value of patient preferences*	*Source*
Understanding the disease	To improve regulators' understanding of the disease.	RE
Benefit-risk assessment	Patient preferences could become part of the different sections of the benefit-risk assessment and could influence the final decision.	IN, RE
*Meaning of benefit-risk profile to patients*	To understand what patients think about certain benefit-risk profiles.	RE
*Maximum acceptable risk assessment*	To assess “*The clinically meaningful benefit for a patient, so maximum acceptable risk to a patient*” (RE4_US).	RE
*Weighing of endpoints*	To explore how important different endpoints are to patients.	RE
*Subgroup identification*	To identify and understand subpopulations with different risk tolerances.	RE
Major contribution to patient care assessment	To understand “*advantage based on a major contribution, so an advantage for a patient*” (RE5_EU) that does not relate to efficacy or safety.	RE
Post-authorization assessments	To re-evaluate the product.	RE
Indication expansion	To support expansion of the approval to other indications.	RE

IN, industry focus group; RE, regulatory focus group.

#### Position of Patient Preferences in HTA

While HTA representatives discussed how PPS would always be considered when submitted, their use in assessments would be on a “*case-by-case basis*” (HTA3_EU). Their incorporation in HTA is uncertain since current frameworks form an operational barrier as they do not give weights to the different decision criteria and are holistic. One HTA representative mentioned that in the future it may be possible to incorporate all aspects in a multi-criteria decision analysis (MCDA). There were differing opinions on whether PPS should be kept separate from other processes and assessments in HTA. HTA representatives expressed the concern that patient preferences could complicate decision-making and could be confusing; they emphasized that use of patient preferences has to be practical and has to fit within their timelines. HTA representatives emphasized that if PPS would be submitted, the objective of the study and how this could facilitate, improve or complicate decision-making should be clear. To allow for assessment of the quality and appropriateness of PPS, HTA representatives thought a tool (checklist or decision tree) could be valuable. However, they acknowledged that assessing quality may be context-dependent, making it difficult to create such a tool. Ideally, the tool would allow for the assessment of sample and method appropriateness, validity, consistency and quality of data. HTA representatives further discussed the different potential applications of patient preferences along HTA processes (**Box 4**). HTA representatives thought patient preferences could be included in cost-effectiveness analyses, but were unsure of the mechanism to enable this other than weighing clinical outcomes and QALYs according to the preferences.

Box 4 **Potential applications of patient preferences in HTA.**

**Table:** 

*Application*	*Value of patient preferences*	*Source*
Scientific advice	To assess if activities in “*development labs*” (HTA4_CAN) are justified.	HTA
Identification of endpoints	To assess which endpoints to consider in assessments.	HTA
Weighing of endpoints	To weigh “*efficacy versus safety*” (HTA3_EU) and “*weigh the end points according to the preferences expressed in preference studies*” (HTA1_EU).	HTA
Subgroup identification	To understand to what subgroups “*the treatment might have particular value within a much larger patient population*” (HTA2_EU), and to understand what the uptake would be in that subgroup.	HTA
General assessment	To use patient preferences in effectiveness assessments to understand the importance of the effectiveness to patients, leading to inclusion of the results in the general assessment of the intervention.	HTA
Cost-effectiveness analysis	To give a weight to clinical outcomes and QALYs, predict real-world adherence and cost-effectiveness.	HTA

HTA, health technology assessment focus group; QALY, Quality Adjusted Life Year.

## Discussion

**Figure 2:**
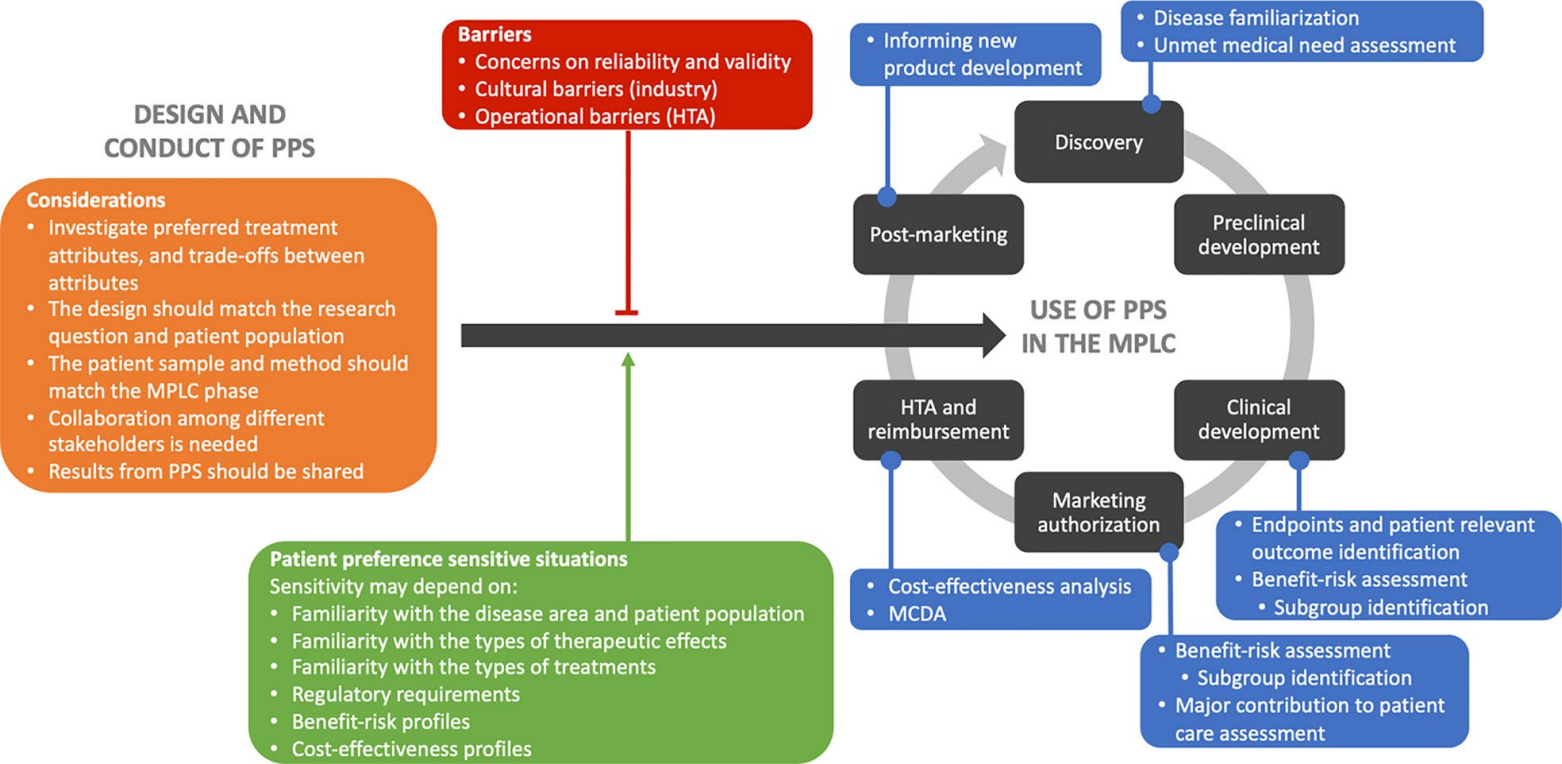
Graphical summary of the main results from the different steps in the multi-method study. Main learnings to consider in the design and conduct of patient preference studies are shown in orange. Barriers to the use of patient preferences in the medical product life cycle are indicated in the red box. In green, the situations sensitive to patient preferences are highlighted as facilitators toward to use of patient preferences. In blue, the applications of patient preferences are indicated throughout the medical product life cycle. Abbreviations: HTA, health technology assessment; MCDA, multi criteria decision analysis; MPLC, medical product life cycle; PPS, patient preference studies.

We performed a multi-method study consisting of literature reviews, interviews and focus groups, to investigate how PPS should be designed, conducted and used in decision-making. The results across the different steps in the study are graphically summarized in **Figure 2**.

### Design and Conduct of Patient Preference Studies

Five main stakeholder beliefs were identified: 1) industry and regulators were interested in PPS showing trade-offs between treatment attributes, and HTA and regulators were interested in what attributes patients prefer; 2) all stakeholder groups wanted PPS to be designed considering the research questions and patient population to ensure understanding of the questions to patients; 3) the MPLC phase was believed to influence patient sample requirements and the choice of the method, as the patient sample should be heterogeneous in early drug development stages where qualitative methods can be applied, and representative of the patient population that is targeted with the therapy in quantitative studies during late development; 4) collaboration among different stakeholders is needed in the design and conduct of PPS, including patients to ensure comprehensibility of questions to patients; and 5) results from PPS should be shared with relevant stakeholders to prevent duplication of efforts and research fatigue in patient populations, and to ultimately inform development of medical products.

In our previous literature review and interview papers on factors to consider in PPS design and conduct (van Overbeeke et al., 2019; Whichello et al., 2019), we identified 15 main factors that can influence the value of PPS and can occur along design and conduct of the studies: expertise, financial resources, study duration, ethics and good practices, patient centeredness, examining patient and/or other preferences, ensuring representativeness, matching method to research question, matching method to MPLC stage, validity and reliability of the method, cognitive burden, patient education, attribute development, patients' ability/willingness to participate, and preference heterogeneity. The focus group results confirm the necessity of different expertise, as also described by Wolka et al. (van Til and Ijzerman, 2014), the Medical Device Innovation Consortium (MDIC) report (MDIC, 2015), van Til et al. (2014), Selig (2016), and Ho et al. (2016). Moreover, these results give further insights on what and why collaborators should be involved, including patients to create a patient centered design, and indicate that PPS results have to be shared among stakeholders. These focus group results give also more insights into what research questions PPS should answer, moving beyond the previous discussion on when patient and when public preferences need to be sought (van Overbeeke et al., 2019; Whichello et al., 2019), and clarifying that PPS should investigate preferred attributes, and trade-offs between benefits and risks, and between benefits and administration attributes. The results of the focus groups also confirm that stakeholders are concerned about the rigor, validity and reliability of PPS (MDIC, 2015; Selig, 2016; Mühlbacher et al., 2017; Janssens et al., 2019; Janssens et al., 2019). The necessary match between the research question, MPLC phase, sample and method was previously described in our literature and interview papers (van Overbeeke et al., 2019; Whichello et al., 2019) and by the FDA (FDA, 2016), Egbrink and Ijzerman (2014), and Gutknecht et al. (2016). However, the focus group results clarify what research questions need to be answered, what samples and methods are useful in what stages of the MPLC, how representativeness of the sample to the population can be ensured and that the method should be matched to the abilities of the patient population to reduce cognitive burden and ensure understanding of questions.

### Use of Patient Preference Studies

Besides the identified stakeholder beliefs, this study also gave insights into how patient preferences can be used in the MPLC. While stakeholders agree that including patient preferences in decision-making is valuable, the submission of PPS and the use of study results in decision-making are limited. Cultural and operational barriers seem to exist that limit their use, mainly among industry and HTA, as previously identified in our interviews (Janssens et al., 2019;Whichello et al., 2019).

Previous studies identified decision-making situations sensitive to patient preferences (van Overbeeke et al., 2019; Whichello et al., 2019), and the focus group results provide a rationale for why that is the case (Box 1). We can conclude that the value of PPS in decision-making depends on the sensitivity of decisions regarding medical products to patient preferences. Sensitivity to patient preferences can relate to the level of understanding and familiarity with the disease area and patient population, the types of therapeutic effects, the types of treatments, regulatory requirements, benefit-risk profiles, or cost-effectiveness profiles.

Four applications along the MPLC were identified that are most promising for the systematic integration of patient preferences in decision-making. First, patient preferences could be used in benefit-risk assessment by attaching weights to endpoints, identifying subpopulations with different risk tolerances, assessing value of products to patients, and assessing maximum acceptable risk. These results are supported by Jackson et al. (2019), Hollin et al. (2017), and Hauber et al. (2013). Second, they can be used in the assessment of major contribution to patient care by European regulators and will improve understanding the value of non-clinical advantages to patients. Third, patient preferences could be used in cost-effectiveness analysis at the HTA stage by weighing clinical outcomes and QALYs, and predicting real-world adherence. Lastly, multi-criteria decision analysis could be applied in HTA by weighing endpoints according to the preferences, as also stated by Mott (2018). On the use of patient preferences in HTA, the HTA representatives seemed to have different opinions regarding whether and how results of PPS need to be integrated. Kievit et al. (2017) created a six question checklist that may meet HTA representatives' needs for a tool to appraise PPS. In addition to these four applications, other important applications identified throughout the three steps of our multi-method study are assessment of disease familiarization and unmet medical needs during the discovery phase, identification of endpoints and patient relevant outcomes during clinical development, and informing new product development in post-marketing phases (MDIC, 2015; FDA, 2016; Selig, 2016; Bloom et al., 2018; van Overbeeke et al., 2019; Whichello et al., 2019; Stamuli et al., 2017).

### Strengths and Limitations

The design of the focus group guides was informed by previous research, and the focus groups were able to dig deeper into topics on which opinions differed during interviews and topics that needed more explanation. Moreover, the topics for the focus group guides were ranked and selected with the help of the relevant stakeholder groups. Overall, in the design, conduct, and analysis of focus groups, a structured and transparent approach was taken.

Although focus groups by nature provide subjective evidence that may not be generalizable to every organization or country, our study ensured the inclusion of diverse types of stakeholders to represent different perspectives. Patients, caregivers and physicians were recruited from different countries and in different disease areas, leading to broad and diverse patient and caregiver samples with different levels of involvement in healthcare. However, we noticed that most patients and caregivers that participated were over 60 years old and had no diploma or a high school diploma, making the results from patient focus groups not representative for younger and higher educated populations. The participants in other focus groups were recruited *via* PREFER contacts, possibly resulting in a sample that was more aware of the concept of patient preferences than the average stakeholder.

Focus groups with industry, regulators and HTA representatives were moderated by two researchers, of which one attended all focus groups. Patient focus groups were moderated by four independent researchers, which may have led to some variability. However, before the start of the patient focus groups all moderators had a meeting to discuss the guide in detail to minimize this variability. As patient focus groups were conducted in the native language of the patients, the materials had to be translated from English to these respective languages. Back-and-forth translation was used to ensure the meaning of the information and questions in the guides and surveys was consistent. Materials were corrected if the back translation showed a different meaning from the original.

### Future Efforts to Improve Implementation of Patient Preferences

The use of PPS in decision-making seems to be in a transition period. If we apply our results to the phases of change management (Barrow and Toney-Butler, 2019), we see that most stakeholders are going through the 'knowledge and persuasion' phases towards the 'decision' phase. In these early phases it is crucial to have change champions (i.e., players that believe in the change and drive the implementation further), like the FDA in the regulatory context and the National Institute for Health and Care Excellence (NICE) from the UK in HTA (FDA, 2016; NICE. NICE provides first scientific advice on patient preference study design, 2019). Through our study we identified multiple issues to be resolved in the preference research area. Future research should focus on quantifying the importance of concepts identified in this study, but also on when and how to use what preference exploration or elicitation method, how to calculate minimum sample sizes considering representativeness requirements for each of these methods, and what weight patient preferences should be given in decision-making. To further advance the implementation of patient preferences in decision-making we also believe it is crucial to pay attention to the 'knowledge' phase of stakeholders and to educate industry, regulators, HTA and payers on how PPS are conducted, what methods can be used and what the possible outcomes of PPS can be. In addition, ways to use patient preferences have to be further explored with educated stakeholders to optimize their implementation in decision-making. To advance the use of patient preferences in HTA and payer decision-making specifically, we believe country or healthcare system-specific studies are needed, as the implementation may be different across systems, to investigate how patient preferences can be incorporated in processes like cost-effectiveness analysis and multi-criteria decision analysis.

## Conclusions

When designing and conducting PPS to inform decision-making in the MPLC, five key considerations need to be taken into account: 1) preferred attributes, as well as trade-offs between benefits and risks, and between benefits and administration attributes should be investigated; 2) the design should be informed by the research questions and patient population; 3) the patient sample and method should match the MPLC phase; 4) collaboration between different stakeholders in needed; and 5) results from PPS should be shared among relevant stakeholders. Moreover, decision-situations sensitive to patient preferences were identified. The more sensitive a decision is, the more value PPS could have. While PPS are not often submitted to regulators and HTA bodies and their use in decision-making is currently limited, potential applications for PPS where identified across industry, regulatory and HTA processes. Some of these applications hold the potential for systematic integration of patient preferences in decision-making in the MPLC. However, stakeholders need to be further educated on the concept of PPS to support efforts exploring how to optimize inclusion of patient preferences in decision-making.

## Data Availability Statement

The datasets generated for this study will not be made publicly available. Participants did not provide consent for the sharing of focus group transcripts with parties other than the researchers.

## Ethics Statement

All participants provided written informed consent. Ethical approval for the multi-method study was obtained from all relevant ethics committees (**Supplementary Material IV**), and the combined design was approved by the Medical Ethics Committee of UZ KU Leuven/Research in Belgium (S59790).

## Author Contributions

EO, RJ, CW, KB, SS, JK, JJ, BL, EB-G, JV, and IH designed the focus group guides. Recruitment and planning of the focus groups was organized by EO, RJ, KSB, JS, NN, PG, GP, LV, GM, and IC. In the conduct of the focus groups EO, RJ, KB, JS, NN, BP, PG, LV, GM, and IH had moderating or assisting roles. The analysis was conducted by EO and RJ. EO produced the first draft of the manuscript, which was subsequently revised and finalized with all authors. All authors approved the final manuscript.

## Funding

The Patient Preferences in Benefit-Risk Assessments during the Drug Life Cycle (PREFER) project has received funding from the Innovative Medicines Initiative 2 Joint Undertaking under grant agreement No 115966. This Joint Undertaking receives support from the European Union's Horizon 2020 research and innovation programme and EFPIA. This text and its contents reflect the PREFER project's view and not the view of IMI, the European Union or EFPIA.

## Disclaimer

This text and its contents reflect the PREFER project's view and not the view of IMI, the European Union or EFPIA.

## Conflict of Interest

JJ declares the following competing interests: employee of Sanofi, a global biopharmaceutical company focused on human health; and ownership of shares in Sanofi and in a portfolio that at times includes other pharmaceutical and health care-related companies. BL declares the following competing interests: employee of Janssen Research and Development, LLC; and stockholder in Johnson & Johnson and in a portfolio that at times includes other pharmaceutical and health care-related companies. JK declares the following competing interests: representing CSL Behring on IMI PREFER; Scientific Consultant working for the pharmaceutical industry; and stockholder in a portfolio that includes pharmaceutical and health care-related companies. BP declares the following competing interests: employee of Pfizer Ltd; and Pfizer share owner.

The remaining authors declare that the research was conducted in the absence of any commercial or financial relationships that could be construed as a potential conflict of interest.
