# Supplementary material for: Design, Conduct, and Use of Patient Preference Studies in the Medical Product Life Cycle: A Multi-Method Study
Source: Front Pharmacol. 2019 Dec 3;10:1395. doi: 10.3389/fphar.2019.01395 (PMC6902285; doi:10.3389/fphar.2019.01395)
Supplement: Supplementary file 1 [file DataSheet_1.pdf]

Design, Conduct and Use of Patient Preference Studies in the Medical Product Life Cycle: a Multi-method Study - Supplementary Material

SUPPLEMENTARY MATERIAL I – FOCUS GROUP GUIDES

I.I Patient Focus Groups

I. Preparations

1. During recruitment
- Aim for 6 participants, 10 participants maximum
- Inclusion criteria:
    - 18y or older
    - Ability to participate in a focus group
    - Country specific criteria:
        - UK: Patient, or informal caregiver of patient diagnosed with myotonic dystrophy. Sample composition: patients and caregivers, but as much patients as possible
        - SE: Patient diagnosed with rheumatoid arthritis. Sample composition: aim for heterogeneous sample in terms of time till diagnosis
        - IT: Patient receiving treatment for, or survivor of lung cancer. Sample composition: 50/50 ratio of patients and survivors
        - RO: Patient diagnosed with cardiovascular disease. Sample composition: aim for heterogeneous sample in terms of time from diagnosis
- Exclusion criteria:
    - Participants that were already interviewed in semi-structured interviews
    - Multiple patients from one family (but if in the end not enough patients are included, siblings can still be welcomed)
- Book a venue/ meeting room that is neutral (hotel, patient organization, or university, not company or government), easily accessible to patients and that allows for a circle seating around a table
- If possible, provide the information sheet to all participants in advance via email a week in advance (otherwise only before focus group on paper)
- Provide the participants with instructions to get to the venue and room
- Provide your/assistants' phone number(s) to the participants

2. Day of the focus group
- Have enough information sheets, consent forms and surveys[1] with you
- Seating arrangements: circle seating around table
- Provide writing material and paper
- Set up the PowerPoint presentation (bring laptop)
- Provide name cards (mentioning only the first name of participants), to put on the table
- Provide water/coffee/tea + something to eat
- Take two audio recorders (+ extra batteries) with you and place these on the table at opposite sides
- Dress code for moderator and assistant(s): Business casual, pants and blouse is OK, no suits, no suit jackets/blazers, not too formal

[1] Health literacy questions: Chew et al. 2008

3. Moderator and assistant team

- A meeting between moderator and assistant should be planned before the focus group to go over the guide and discuss the moderator and assistant's tasks

3.1 Attitude of moderator:

- Exercise mild unobtrusive control (moderate the discussion but do not interrupt too often)
- Adequate knowledge of topic
- Appears like the participants
- Use purposeful small talk
- Alert and free from distractions (put sound of phone off and do not have your phone on you)
- Have the discipline of listening and apply active listening
- Familiar with questioning route (know this protocol, the timing of slides + animations in slides and all questions very well)
- Take into account the different types of participants and try to balance the conversation while addressing the obligatory topics: dominant talkers, shy participants, etc.

3.2 Tasks of the assistant:

- Handles logistics (location of refreshments, bathrooms, emergency exits)
- Collects consent forms and surveys
- Takes careful notes (bring laptop)
    - On statements made
    - Nonverbal reactions (movements, attitudes, emotions)
- Controls for equal participation by all participants and informs the moderator if some participants are not getting the chance to participate
- Monitors audio recording equipment
- Time management via discrete signs to the moderator
- Gives a general summary of what has been said at the end of the focus group

II. Focus group agenda

Everything below in *Italic* is to be said to the participants, everything in black is guidance and if applicable can be told to the participants in your own words. In **bold** the timing of actions is given; however, this is an indication of time, it is more important to finish the two first topics than to rush through the focus group and complete all three topics. In **bold** also, the slides to display is given.

4. **00:00 Slide 1**. Welcome the participants while they arrive
   - Create warm and friendly environment
     - Provide coffee and tea
     - Interact with participants, and stimulate interaction between participants
   - Make seating arrangements for participants according to their needs
   - Provide the information sheet and let them fill in the consent form and survey

5. **00:06 Slide 2**. When all participants arrived and completed the forms, start the focus group with a general introduction:
   - *Welcome, my name is* (your first name) *and I will be your moderator today. In addition, I brought* (first name of assistants) *to help me with the focus group*
   - *My role as moderator will be to guide the discussion*
   - *The discussion that we will have today is on your involvement, or actually your preferences and how these preferences can be used in making drugs or medical devices, and in making them available to you*
   - *Today's discussion is part of a large European project with similar objectives*
   - *We want to have this discussion with you as patients (and caregivers), since we think it is important to know how you feel about using your preferences*
   - *The opinions collected today will be used to write reports and publications to inform companies, health authorities, researchers and other parties on how to use patient preferences*
   - *The focus group will take 1 hour to 1.5 hours*
   - *There will be a break in the middle of the discussion of 5-10 minutes*

6. **00:08**. Explain the rules:
   - *There are no right or wrong answers, only differing points of view*
   - *We are looking for your opinions and hope for a nice discussion*
   - *It is possible that you do not agree with all opinions, but it would be nice if you could listen respectfully to each other*
   - *We're audio recording, so it would be very helpful for the analysis if only one person is speaking at a time*
   - *Since this is an informal discussion, we will address each other only by their first name as indicated on the name cards*
   - *We ask you to turn off the sound of your phones*
   - *If there are any questions or terms that are used during the focus groups that are not clear to you, please let us know*
   - *So, we will now start the recording. Is that OK for everybody?*
   - *First, to get to know each other, could everybody state their name and the city or village he/she is from?*

7. **00:10 Slide 3**. Introduce the topic of patient preferences:
   - Definition: *In patient preference studies, we want to examine why patients choose a particular health treatment over other available options. This health treatment can be a drug or a medical device. A preference can be stated for a health treatment as a whole or for the advantages and disadvantages of one treatment. In order to make a choice or state a preference, patients need to weigh up the advantages and disadvantages and compare them to those of other health treatments.*

- **Slide 4**. Example: *A patient preference study asks patients about what they want from a flu vaccine (flu shot) by asking what different characteristics, here represented by the different colors, matters most to them, such as if it works well, how long it lasts, its side-effects, where patients can get it, or how much it costs. Some patients might say that the effectiveness of the vaccine is most important, while others think that a vaccine should have the lowest level of side-effects. Therefore, we ask patients to fill in a survey or take part in an interview for example, to in the end see what matters the most to each patient.*
- **Slide 5**. *Patient preferences can be used by companies or the government in making drugs and making them accessible to patients*
- Ask if this is understandable to all
- If there are any questions or terms that are used during the focus groups that are not clear to you, please let us know

8. **00:15 Slide 6**. Introduce the first question:
   - *Different things may influence what you prefer, and this is of course something that we want to examine in patient preference studies. Therefore, we would like to know:*
     - ➢ ***What are the factors that you consider when making a choice between treatments?***
       - o *Alternatives:*
         - ▪ *What factors will influence your preference?*
         - ▪ *What matters to you when you have to make a choice?*
         - ▪ *What do we need to measure in patient preference studies?*
   - Obligatory topics to address:
     - o Which characteristics of *treatments* should be examined (examples: benefits, risks, advantages, disadvantages, route of administration, frequency of administration, geographical place where the drug can be taken or injected)
     - o Which characteristics of *participants* should be taken into account (examples: education, finances, cognitive ability, risk taking, medical need)
   - *So now we know a little bit about what we need to look for in these studies. Then I would like to ask:*
     - ➢ ***What is the best way to ask about your preferences?***
   - Obligatory topics to address:
     - o What type of questions to ask (examples: general versus disease specific questions)
     - o Survey, interview or other manners
     - o Information need and capability to indicate preferences (examples: what information on the product/drug life cycle/research/etc. is needed for patients to feel like they can give an informed useful answer)
   - Summarize with confirmation
   - Review purpose and ask if anything has been missed
   - Thank and move on to next topic

9. **00:30 Slide 7**. Have a break of 5-10 minutes.

10. **00:40 Slide 8**. Introduce the second question:
    - *When doing a patient preference study, we of course need to search for patients that are willing to participate. Sometimes it is hard to find patients that want to participate. Therefore, we would like to know from you:* **animation 1**
      - ➢ ***Why would you <u>want</u> to participate?***
    - Obligatory topics to address:
      - o Incentives to participate (examples: advancement in drug research, hope for faster access, to give patients a voice, remuneration, interest, etc.)

    - *Then we would also like to examine participation from another perspective, and we would like to know from you:* **animation 2**

- ➢ ***Why would you <u>not</u> want to participate in a patient preference study?***
- Obligatory topics to address:
  - o Barriers to participation (examples: not interested, does not see the need, thinks the preferences will not have an influence, difficulties in getting to research site, financial issues, data privacy)
  - o Possible solutions (examples: solutions to barriers mentioned above)
- Summarize with confirmation
- Review purpose and ask if anything has been missed
- Thank and move on to next topic

11. **00:55 Slide 9**. Introduce the third and final question:
    - *Different organizations involved in health care can perform patient preference studies. So, these studies will not only be conducted by universities, but for example also pharmaceutical companies, health authorities, or patient organizations.*
      - ➢ ***What do you think about this?***
    - Obligatory topics to address:
      - o Handling the data (examples: How should these organizations handle your data?)
      - o Data sharing (examples: Do you think they can share the data between universities, or between universities and companies? Is this different for your preferences than for information about your disease or treatment?)

12. **01:10 Slide 10**. Finish
    - Summarize the lessons learnt of today's discussion – by assistant
    - Ask if the summary is correct, or if you have forgotten something
    - Ask if there are any questions
    - **Slide 11**. Thank all participants for their participation – finish

The focus group is now finished

13. Collect the following materials and put them on Sharepoint:
    - Consent forms
    - Short surveys
    - Recordings
    - Notes

I.II Regulatory, HTA and industry Focus Groups

I. Preparations

1. During recruitment
   - Having 6 participants is a minimum, aim for 8 participants
   - Inclusion criteria:
     - Experienced professionals suggested by EFPIA, HTA SAG PREFER, EMA or FDA contact persons.
     - Stakeholder specific criteria:
       - Industry: Working for a pharmaceutical company. Person should be based in Europe. Sample composition: 1-2 persons per category R&D, regulatory affairs, market access, and patient engagement.
       - Regulators:
         - EMA: Working for EMA.
         - FDA: Working for the FDA. Sample composition: Balanced sample of people working in CDER, CBER and CDRH.
       - HTA Working for a European HTA body. Sample composition: HTA/payer Advisory Group of PREFER.
   - Exclusion criteria:
     - Persons already interviewed in the semi-structured interviews.
   - Plan and set up a teleconference meeting with all participants via Skype for Business
     - Provide the information sheet and consent form to all participants in advance via email and ask participants to sign and send back the consent form a day before the focus group (send reminder a week before the focus group)
     - Ask participants to call in separately, alone, in a quite space and with their camera and sound on
   - Provide your and your assistants' phone number(s) to the participants

2. Day of the focus group
   - Check if you received signed informed consent forms of all participants. If participants did not sign their consent form, they cannot participate in the focus group
   - Have two audio recorders (+ extra batteries) with you and place these near your laptop
   - Dress code for moderator and assistant(s): Business, formal

3. Moderator and assistant team

- A meeting between moderator and assistant should be planned before the focus group to go over the guide and discuss the moderator and assistant's tasks

3.1 Attitude of moderator:

- Calls into the teleconference separately from assistants via telephone, not computer
- Exercise mild unobtrusive control (moderate the discussion but do not interrupt)
- Steering the focus group where needed
- Adequate knowledge of topic
- Appears like the participants
- Use purposeful small talk
- Do not complement or discourage stakeholders on the points they make
- Alert and free from distractions (put sound of cell phone off and do not have your cell phone on you)
- Have the discipline of listening and apply active listening
- Familiar with questioning route (know this protocol very well)
- Take into account the different types of participants and try to balance the conversation while addressing the obligatory topics: dominant talkers, shy participants, etc.

3.2 Tasks of the assistant:

- Calls into the teleconference separately from the moderator and other assistants via telephone, not computer
- Takes careful notes (bring laptop)
    - Verbal reactions (Audio: Discussion, changes in attitude (voice)
    - Nonverbal reactions (Video: movements, attitudes, emotions)
- Controls for equal participation by all participants and informs the moderator if some participants are not getting the chance to participate
- Monitors audio recording equipment
- Time management via discrete signs or verbal communication to the moderator
- Gives a general summary of what has been said at the end of the focus group

II. Focus group agenda

Everything below in *Italic* is to be said to the participants, everything in black is guidance and if applicable can be told to the participants in your own words. In **bold** the timing of actions is given; however, this is an indication of time, it is more important to finish the two first topics than to rush through the focus group and complete all three topics.

4. **00:00** Welcome the participants while they call in on the teleconference
   - Try to get every participant's name directly and welcome them
   - If participants try to participate without having signed the consent form, ask them if they can sign the consent form on spot and send a scan per e-mail within a couple of minutes. If this is not possible, ask them to leave the teleconference
   - Wait until all participants have joined for 5 minutes from start of teleconference

5. **00:06** When all participants arrived and completed the forms, start the focus group with a general introduction:
   - *Welcome, my name is* (your first name), *working as PhD researcher at* (institute) *and I will be your moderator today. In addition, also* (first name of assistants) *called in to help me with the focus group*
   - *My role as moderator will be to guide the discussion*
   - *The discussion that we will have today is on the assessment and use of information on patient preference studies when making decisions during the development of drugs or medical devices, and when making them available to patients*
   - *Today's discussion is part of a study within a large European project called IMI PREFER. PREFER looks at how and when it is best to perform and include patient-preference in decision making during the drug life cycle. The end-result will be recommendations to support development of guidelines for industry, Regulatory Authorities and HTA bodies.*
   - *We want to have this discussion with you as (name stakeholder), since we think it is important to know how you feel about using these preferences in the decision making at your institution(s)*
   - *The opinions collected today will be used to write reports and publications to inform companies, health authorities, researchers and other parties on how to use patient preferences*
   - *The focus group will take about 1.5 hours*

6. **00:08** Explain the rules:
   - *There are no right or wrong answers, only differing points of view*
   - *We are looking for your opinions and hope for a good discussion*
   - *It is possible that you do not agree with all opinions, listen to each others points of view; each view is important and counts. Afterwards you are welcome to provide your view.*
   - *We're audio recording, so it would be very helpful for the analysis if only one person is speaking at a time*
   - *Since this is an informal discussion, we will address each other only by their first name*
   - *If there are any questions or terms that are used during the focus groups that are not clear to you, please let us know*
   - *So, we will now start the recording. Is that OK for everybody?*
   - *First, to get to know each other, could everybody state their name and job title?*

7. **00:10** Introduce the topic of patient preferences:
   - Definition of the FDA: *"The relative desirability or acceptability to patients of specified alternatives or choices among outcomes or other attributes that differ among alternative health interventions"*
   - *In other words, patient preferences are the basis of how patients choose a particular treatment over others. To make a choice, patients make trade-offs between a treatment's characteristics, weighing its advantages and disadvantages collectively.*

- Example: *A patient preference study asks patients about what they want from a flu vaccine (flu shot) by asking what different characteristics matters most to them, such as the probability that it is effective, how long it lasts, its side-effects, where patients can get it, or how much it costs. Some patients might say that the effectiveness of the vaccine is most important, while others think that a vaccine should have the lowest level of side-effects. Therefore, we ask patients to fill in a survey or take part in an interview for example, to in the end see what matters the most to each patient.*
- Ask if this is understandable to all
- *If there are any questions or terms that are used during the focus groups that are not clear to you, please let us know*

8. **00:15** Complete the focus group by going over the stakeholder-specific questions on the following pages of this protocol.

9. **After completion of step 8, the focus group is finished.**
01:50 Final summary by the assistant, to check if anything was misinterpreted by moderator and assistants
01:55 Ask if anything has been missed, or if anybody would like to add anything
02:00 Thank all participants for their participation and stop the teleconference

Collect the following materials and put them on Sharepoint:

- Consent forms
- Recordings
- Notes

III. Stakeholder-specific questions (step 8)

III.I Industry

1. **00:15** *Representativeness of the patient sample is an important quality indicator of patient preference studies. We would like to explore your understanding on this topic of representativeness. Therefore, I would like to ask you:*

- **What does a representative patient sample mean to you?**
- **How can representativeness of the patient sample be ensured?**

Besides representativeness of the patient sample we would like to know what characteristics, according to you, the patients should have to be able to participate in a patient preference study

- **According to you, what would an ideal patient sample look like?**
  - *How does it depend on the research question being asked?*
- Summarize (very briefly) with confirmation
- Review purpose and ask if anything has been missed
- Thank and move on to next topic

2. **00:45** *Most pharmaceutical companies are trying to engage patients. Patient preferences are sometimes elicited and used in company decision making. However, the use of patient preferences in internal decision making in companies is unstructured and not systematic. In addition, the possibilities and processes to incorporate patient preferences in industry decision making remain unclear. Therefore, I would like to ask you:*

- **How does industry conceptualise patient preferences?**
  - *Through which measures do you believe they should be expressed?*
  - *Do current measures adequately and sufficiently reflect patient preferences?*
- **What do you think about using patient preferences in a more structured way for internal industry decision making?**
  - *Do you think it should be more structured than it is now?*
- **What kind of patient preference studies would be valuable for industry decisions?**
  - **Which research questions should they answer?**
  - If interviewees do not understand question: examples of topics some patient preference studies address are clinical endpoints, PRO tools and their relevance to patients, cost, wtp, burden of disease, social/ethical impact, how patients value the outcomes of the therapy
- **How can patient preferences be integrated in internal industry decision making?**
  - **In which evaluations or processes could this be integrated?**
  - Try to answer this question within the following stages:
    - Early R&D
    - Clinical development
    - Regulatory affairs
    - Market Access
- **What weight should patient preferences receive in internal industry decision making compared to other decision criteria?**
  - Follow-up: *How can patient preferences be compared to, or combined with other decision criteria in internal industry decisions?*
  - Examples of other decision criteria: Efficacy, safety risks, production costs, etc.
- **In what conditions do you think that it is necessary to take patient preferences into account in regulatory decision making?** (+Why)
  - Synonyms for conditions can be situations, circumstances
  - Try to get an answer on:
    - For which patient populations,
    - Types of treatments,
    - Benefits/risks,
    - Other conditions

- Summarize (very briefly) with confirmation
- Review purpose and ask if anything has been missed
- Thank and move on to next topic
- Summarize (very briefly) with confirmation
- Review purpose and ask if anything has been missed
- Thank and move on to next topic

3. 01:30 *The following topic deals with handling unexpected results from patient preferences studies. "Imagine there's an innovative drug being developed. The phase II clinical trial results show the drug has high benefits, but also high risks. A patient preference study in phase III indicates that patients do NOT want to accept the risks."*

- **How do you think most pharmaceutical companies would handle this situation?**
- **What do you think will be the impact of this situation on the further use of the results of the preference study?**
- **How do you think most companies will communicate the results of the preference study?**
    - *Internally, within the company?*
    - *Externally, towards external stakeholders?*
- **How do you think most pharmaceutical companies would handle this situation if the patient preference study shows that patients would accept more risk than anyone designing the study thinks is reasonable?**
- Summarize (very briefly) with confirmation
- Review purpose and ask if anything has been missed
- Thank participants for input

III.II Regulators

1. **00:15** *Representativeness of the patient sample is an important quality indicator of patient preference studies. We would like to explore your understanding on this topic of representativeness. Therefore, I would like to ask you:*

- **What does a representative patient sample mean to you?**
- **How can representativeness of the patient sample be ensured?**

Besides representativeness of the patient sample we would like to know what characteristics, according to you, the patients should have to be able to participate in a patient preference study

- **According to you, what would an ideal patient sample look like?**
  - *How does it depend on the research question being asked?*
- Summarize (very briefly) with confirmation
- Review purpose and ask if anything has been missed
- Thank and move on to next topic

2. **00:45** *The importance of patient preferences in decision making seems to be increasing. However, actual application and integration of patient preferences in regulatory decision making is limited and not systematic. In addition, the possibilities and processes to incorporate patient preferences in regulatory decision making remain unclear. Therefore, I would like to ask you:*

- **How do regulators conceptualise patient preferences?**
  - *Through which measures do you believe they should be expressed?*
  - *Do current measures adequately and sufficiently reflect patient preferences?*
- **What kind of patient preference studies would be valuable for regulatory decision making?**
  - **Which research questions should they answer?**
  - If interviewees do not understand question: examples of topics some patient preference studies address are clinical endpoints, PRO tools and their relevance to patients, cost, wtp, burden of disease, social/ethical impact, how patients value the outcomes of the therapy
- **How do you think that patient preferences could be integrated in regulatory decision making?**
  - **In which evaluations or processes could this be integrated?**
  - Try to answer this question within the following stages:
    - Pre-submission activities
    - Marketing-Authorization
      - Submission & validation
      - Scientific opinion
      - Commission decision
    - Post-authorisation activities
- **What weight should patient preferences receive in regulatory decision making compared to other decision criteria?**
  - Follow-up: *How can patient preferences be compared to, or combined with other decision criteria in regulatory decisions?*
  - Examples of other decision criteria: Efficacy, safety risks, production costs, etc.
- **In what conditions do you think that it is necessary to take patient preferences into account in regulatory decision making?** (+Why)
  - Synonyms for conditions can be situations, circumstances
  - Try to get an answer on:
    - For which patient populations,
    - Types of treatments,
    - Benefits/risks,
    - Other conditions
- Summarize (very briefly) with confirmation
- Review purpose and ask if anything has been missed

- Thank and move on to next topic

3. **01:30** *The following topic deals with stakeholder roles in patient preference studies.*
   - **Who do you think should conduct a patient preference study aiming to inform regulatory decision making?**
     - **What do you think should be the role of the sponsor of the patient preference study in the design of the study?**
     - **What do you think should be the role of the sponsor of the patient preference study in the conduct of the study?**
     - **What do you think should be the role of the sponsor of the patient preference study in the analysis of the study results?**
   - **In what manner do you think that conducting parties can influence patient preference studies?**
   - If the interviewees express this is a concern: **How do you think those influences can be reduced?**
   - Summarize (very briefly) with confirmation
   - Review purpose and ask if anything has been missed
   - Thank participants for input

III.III HTA

1. **00:15** *Representativeness of the patient sample is an important quality indicator of patient preference studies. We would like to explore your understanding on this topic of representativeness. Therefore, I would like to ask you:*

- **What does a representative patient sample mean to you?**
- **How can representativeness of the patient sample be ensured?**

Besides representativeness of the patient sample we would like to know what characteristics, according to you, the patients should have to be able to participate in a patient preference study

- **According to you, what would an ideal patient sample look like?**
    - *How does it depend on the research question being asked?*
- Summarize (very briefly) with confirmation
- Review purpose and ask if anything has been missed
- Thank and move on to next topic

2. **00:45** *The importance of patient preferences in decision making seems to be increasing. However, actual application and integration of patient preferences in HTA and payer decision making is limited and not systematic. In addition, the possibilities and processes to incorporate patient preferences in HTA and payer decision making remain unclear. Therefore, I would like to ask you:*

- **How do HTA/payers conceptualise patient preferences?**
    - *Through which measures do you believe they should be expressed?*
    - *Do QALYs and QoL indexes adequately and sufficiently reflect patient preferences?*
- **What kind of patient preference studies would be valuable for HTA and payer decisions?**
    - **Which research questions should they answer?**
    - If interviewees do not understand question: examples of topics some patient preference studies address are clinical endpoints, PRO tools and their relevance to patients, cost, wtp, burden of disease, social/ethical impact, how patients value the outcomes of the therapy
- **How do you think that patient preferences could be integrated in HTA and payer decision making?**
- Make sure the discussion stays on the use of patient preferences, not public preferences for reimbursement decisions.
    - **In which evaluations or processes could this be integrated?**
        - Early dialogue/scientific advice (to discuss clinical endpoints and PROs)
        - During submissions and reimbursement discussions
        - Post-marketing approval (real world experience?)
        - And would they be more an input to the deliberative process or also to the Economic evaluations: Cost benefit analysis, cost effectiveness analysis, or cost utility analysis?
- **What weight should patient preferences receive in HTA and payer decision making compared to other decision criteria?**
    - Follow-up: *How can patient preferences be compared to, or combined with other decision criteria in regulatory decisions?*
    - Examples of other decision criteria: Efficacy, safety risks, quality, cost, etc.
- **In what conditions do you think that it is necessary to take patient preferences into account in HTA and payer decision making?** (+Why)
    - Synonyms for conditions can be situations, circumstances
    - Try to get an answer on:
        - For which patient populations,
        - Types of treatments,
        - Benefits/risks,
        - Other conditions
- Summarize (very briefly) with confirmation
- Review purpose and ask if anything has been missed

- Thank and move on to next topic

3. **01:30** *The following topic deals with quality requirements for patient preference studies aiming to inform HTA and payer decisions.*

- **What is your opinion on having a standard quality checklist for patient preference studies aiming to inform HTA and payer decisions?**
    - **Do you think that the quality criteria for patient preferences studies for HTA and payer decisions need to be clearer?**
- **What should this quality checklist look like?**
    - **What topics should be addressed in the quality checklist?**
- **Who should conduct a patient preference study aiming to inform HTA and payer decisions?**
- Summarize (very briefly) with confirmation
- Review purpose and ask if anything has been missed
- Thank participants for input

SUPPLEMENTARY MATERIAL II – FOCUS GROUP ANALYSIS PLAN

II.I Steps and rationale behind the choice of the analysis method

The steps we undertook to select our analysis method included the following:

1. First, we reviewed the current analysis methods, consulting published articles, books and web pages on qualitative analysis methods. This review revealed that a variety of methods exist to analyze focus groups:
   - Stalmeijer RE, McNaughton N, Van Mook WN. Using focus groups in medical education research: AMEE Guide No. 91. Med Teach. 2014;36(11):923-39 →This paper gives a rather broad description and refers to other guides: Krueger & Casey, Barbour, Kidd & Parshall 2000, Pope & Mays
   - http://research.apc.org/images/2/2f/A_Qualitative_Framework_for_Collecting_and_Analyzing_Data_in_Focus_Group_Research.pdf → Many types of analysis, constant comparison analysis seems appropriate for multiple focus groups, however we do not want to analyze different stakeholder Focus Group Discussions (FGDs) together, as they cover different topics
   - https://www.discuss.io/blog/focus-groups/4-ways-analyze-focus-groups-reduce-friction/ → Many types of analysis, constant comparison analysis seems appropriate for multiple focus groups, however we do not want to analyze different stakeholder FGDs together, as they cover different topics
   - Focus groups: a practical guide for applied research (2000) - Krueger, Richard A.; forew. by Michael Quinn Patton → Only explains the "long-table approach" (using scissors), not manageable
2. Next, we consulted a qualitative research expert from the University of Leuven (external to the PREFER project), who suggested 2 approaches based upon our research objectives:
   - Multiple case design (Miles and Huberman, 1994) → This approach focusses on performing within and across case analyses to reveal similarities and differences among the different FGDs, which why we thought it was appealing to our study. However, we rejected this approach as it is also a very time consuming one;
   - Thematic analysis (Howitt, 2016) → Selected as our method.

Rationale behind the selection of thematic analysis:

1. Thematic analysis focusses on what has been said rather than how it was said;
2. Thematic analysis is accessible to novice researchers, as it is less demanding than other methods (e.g. thematic analysis does not involve the same level of sophistication in data collection and theory building as grounded theory);
3. Useful technique when:
   a. Data collection has finished; thematic analysis, as opposed to grounded theory, does not have the requirement that the data being collected are reviewed part-way through the analysis and new approaches to data collection are initiated;
   b. Data consists of detailed textual material (e.g. focus group transcripts);
   c. A broad-brush approach to analysis is desired (as opposed to some fine-grained approaches which characterize some qualitative methods);

"How to do thematic analysis" (p.168-175, Howitt, 2016) has formed the basis of this protocol.

II.II Protocol

Chosen method: Thematic analysis based upon “Qualitative Research Methods in Psychology” – Dennis Howitt

Core analyzing team (“CAT”): Eline van Overbeeke (EvO) and Rosanne Janssens (RJ)

Major steps in thematic analysis:

1. Data familiarization
2. Initial coding generation
3. Search of themes
4. Review of themes
5. Theme identification and labelling

Step-wise explanation:

1. Data familiarization

a. Howitt states that:
   i. Data familiarization can take place via several methods and will differ according to the details of the study. Ways of familiarizing with the data are: (i) being involved in the data collection stage, (ii) doing the transcription, (iii) playing the recordings repeatedly or (iv) re-reading the transcripts;
   ii. Start think about what is happening in the data during this stage. These early thoughts may suggest ways in which the data might be coded;
   iii. Use literal transcription (since thematic analysis is about what was said rather than how it was said);
   iv. Novice researchers to do all the data collection and transcription themselves;

b. Therefore, we will:
   i. Become familiar with the data by:
      1. Being present during the FGD as moderator or assistant to the moderator. Because of practical limitations, 2 FGDs were moderated by an external partner, which make the steps below critical to become familiar with those FGDs. These external partners were actively involved during the set-up the research (e.g. during the set-up of the protocol and FGD questions) as we had several discussions with them to inform them and ask about their feedback about our focus group questions and protocol. Next, because of the amount of the focus groups (n=8) and the different languages used, we chose to outsource the transcription and translation of the focus groups to a professional transcription company (*TodayTranslations*).
      2. Thoroughly reading the transcript several times;
   ii. Have the transcription done literally (at verbatim) by the transcription company;
   iii. Have the transcripts checked for completeness and accuracy by moderator and/or assistants present during FGD;
   iv. Convene and discuss in order to agree together about what was said in that particular part of the FGD, should there be difficulties in understanding the transcript. Should there be any remaining difficulties in understanding a certain part of the FGDs where neither one of EvO or RJ was present, we will contact the moderator and/or assistant present during that FGD to discuss and agree upon what was said.

2. Initial coding generation

c. Howitt states that:
    i. This stage does not aim at identifying the themes that the research will generate; initial codes are nothing more than labels that will describe the content of 1 or 2 lines, they are not sophisticated analyses of the data. However, ideas as to what the themes might be can already occur (as during any stage of thematic analysis);
    ii. There are no "rules" describing that initial coding has to be done line-by-line. Coding frequency depends on circumstances, if every line is not possible then every 2/3 lines is "all right";
    iii. Best if these codes are based on an abstraction rather than something concrete, the more conceptual (i.e. the less concrete, the more abstract) the codes, the better the final themes;
    iv. Researchers can choose:
        1. To work through the entirety of data or a subset of the data selected because it deals with a topic/matter of interest to the researcher;
        2. Between a theory-led or data-led approach;
    v. During this stage, it may be appropriate to re-name codes that are covering the same meaning so they have the same wording;
    vi. After the initial coding has been done, researchers should put together all of the transcript which has received a certain code. Reviewing all coded text of a certain code can reveal that:
        1. A coding label is not accurate/precise enough and needs to be renamed;
        2. New codes need to be formed as some of the data in a certain code "does not match";
        3. Certain codes need to be combined to one code as the coded text below two codes is too similar.

d. Therefore, we will:
    i. Aim for 1 initial code every 2/3 lines;
    ii. Use a data-led approach as described by Howitt, in which codes are primarily guided by careful analysis of what is in the data;
    iii. Independently code the entirety of the data (n=8 FGDs), since there are two core analyzers;
    iv. Convene after the independent coding of the data and perform steps vi.1, 2 and 3 together in order to agree upon the final list of initial codes

3. Search for themes based on initial codes

e. Howitt states that:
    i. This stage involves turning the initial codes into themes, which requires a lot of analytic work on the part of the researchers;
    ii. Searching for themes involves searching for patterns among the initial codes; as they will probably notice that some codes are more related than others;
    iii. Themes are the result of grouping and categorizing codes, which does not preclude that some codes might turn out to be very important and result in this code being an actual theme;
    iv. Some themes may be very obvious from the initial codes, whereas sometimes methods of sorting might help, e.g. by writing down all the initial codes on separate slips of paper and creating piles of related codes. NVivo or Word might be used in this stage.

f. Therefore, we will:
    i. Each independently search for themes based upon the initial codes;
    ii. Convene, discuss and agree upon the themes that we independently found.

4. Review of themes

g. Howitt states that:
    i. At this stage, there is a set of tentative themes which help to understand the data;
    ii. In the case that these themes are not fully defined or refined at this stage, it is essential to examine these themes against the original data;
    iii. Reviewing of themes involves organizing the data around the set of themes just as previously the data was organized around the codes;
    iv. The possible scenarios of this stage are:
        1. Modifying or abandoning the theme if there is very little in the data supporting the theme;
        2. Dividing or subdividing the theme if the data in one theme actually imply two different themes or sub-themes;
        3. Find a new theme if some of the data you initially believed were part of the theme does not fit. If this is the case, a check for applicability of these themes to this data as well as the entire data set is advised.
h. Therefore, we will:
    i. Divide the total number of transcripts among EvO and RJ;
    ii. Separately go back to each of the assigned transcript and organize the text that was captured by the initial codes around our identified themes;
    iii. Separately critically revise whether the theme should be abandoned, modified, (sub)divided or whether a new theme should be found;
    iv. Any modification to our initial found themes will trigger a discussion between EvO and RJ and should this discussion lead to a modification of the initial list of themes, a check of the applicability of this modified list of themes to the entire data set will be done.

5. Theme definition and labelling

i. Howitt states that:
    i. Although it might be easy to give a label to a theme, it might be more difficult to define exactly what a theme is;
    ii. It is important to be able to conceptually distinguish one theme from another;
    iii. It is likely to continue developing sub-themes at this stage;
    iv. It is important to talk with other people about your analysis at this stage and allow them to question you and throw in ideas of their own.
j. Therefore, we will:
    i. Discuss and agree upon the final list of themes and sub-themes and our explanation to it. This will form the basis of the report (see 6.);
    ii. Discuss within our CAT as well as with other persons how our themes differ from each other.

6. Report writing

k. Howitt states that:
   i. The explanation and description of the themes in the final report of thematic analysis involve the selection of appropriate illustrations taken from the material which is associated with the theme;
   ii. Criteria that may be applied for this selection are:
      1. How ‘typical’ the material is of the data which belong to a particular theme;
      2. How ‘fit’ the material is in relation to the theme; some excerpts might illustrate particular features of the theme better than others;
      3. How ‘eye-catching’ the excerpt is; some data might be preferred to other excerpts as it is more vivid;
      4. Some might prefer using excerpts from just one of the participants to get into more detail about that particular case
   iii. It is helpful to indicate in the report the basis for your excerpt selection

l. Therefore, we will:
   i. Explain and describe in the final report the themes we identified;
   ii. Use appropriate excerpts to illustrate these themes;
   iii. Apply criteria ii.1, 2, 3 to select the excerpts we will use to describe the themes.

SUPPLEMENTARY MATERIAL III – LIST OF FINAL THEMES AND DEFINITIONS

III.I Patient focus group themes

Themes	Definition
1. Healthcare issues that matter to patients	All topics, issues, concerns, desires, needs, preferences important to patients in relation to their or others' healthcare
1.1 Medical product related issues	All topics, issues, concerns, desires, needs, preferences important to patients in relation to medical products and their characteristics
1.2 Non-medical product related issues	All topics, issues, concerns, desires, needs, preferences important to patients not directly in relation to medical products and their characteristics, but still relating to healthcare
1.2.1 Patient journey related issues	All topics, issues, concerns, desires, needs, preferences important to patients relating to all the sequential steps in receiving healthcare
1.2.1.1 Diagnosis related issues	All topics, issues, concerns, desires, needs, preferences important to patients relating to their diagnosis
1.2.1.2 Individual treatment decision making related issues	All topics, issues, concerns, desires, needs, preferences important to patients relating to individual treatment decision making
1.2.1.3 Psychological support	All topics, issues, concerns, desires, needs, preferences important to patients relating to the support they receive of others
1.2.2 Healthcare system related issues	All topics, issues, concerns, desires, needs, preferences, points of critique, frustrations relating to the healthcare system including drug development, regulators, access, reimbursement/financing, healthcare budget, etc.
2. Healthcare issues that matter to caregivers	All topics, issues, concerns, desires, needs, preferences important to caregivers in relation to their or others' healthcare and quality of life
3. Designing and conducting patient preference studies	Topics highlighted by patients and caregivers to consider in designing and conducting patient preference studies
3.1 Participant characteristics	The type of participants that should be included in patient preference studies according to patients and caregivers
3.2 Methodology	Considerations for the methods and instruments in patient preference studies according to patients and caregivers
3.3 Participants' information needs	The type of information patients and caregivers would like to receive before or when participating in a patient preference study
3.4 Factors influencing participation	Incentives, barriers, facilitators, hurdles that influence whether or not patients would like to participate in patient preference studies
3.5 Handling patient preference study data	The manner by which patient preference study data should be managed according to patients and caregivers taking into account privacy, confidentiality, data sharing and access

III.II Industry focus group themes

Themes	Definition
1. Designing, conducting and reporting patient preference studies	Topics highlighted by industry representatives to consider in designing, conducting and reporting on patient preference studies
1.1 Research questions	The questions and topics that industry representatives hope patient preference studies will answer and the patient preference information they would find valuable to know
1.2 Sample considerations	Factors to take into account when determining whose preferences should be measured
1.3 Methodology	Considerations for the methods and instruments in patient preference studies according to industry representatives
1.4 Organizational considerations	Considerations for the methods and instruments in patient preference studies relating to budget, duration and timing along the medical product life cycle
1.5 Stakeholder roles in patient preference studies	Stakeholder responsibilities and collaborations in patient preference studies according to industry representatives
1.6 Rigour, validity and robustness of patient preference studies	How rigourous, valid and robust patient preference studies should be, aiming to inform decision-making of evaluators
2. Use of patient preferences in industry decision-making	What do industry representatives think about using patient preferences in industry, including their value, the methods to measure them and the potential application of patient preferences in industry
2.1 Value of patient preferences in industry	The (added) value of patient preferences in industry processes and factors or situations influencing this value
2.2 Current conceptualization of patient preferences in industry	How are patient preferences currently captured in industry processes
2.3 Position of patient preferences in industry	How do industry representatives see the use of patient preferences from (conducted) patient preference studies
2.4 Remaining questions	Questions industry representatives raised but could not answer related to the use of patient preferences in their processes
3. Applications of patient preferences outside industry	What do industry representatives say about using patient preferences by regulators and HTA

III.III Regulatory focus group themes

Themes	Definition
1. Designing, conducting and reporting patient preference studies	Topics highlighted by regulatory representatives to consider in designing, conducting and reporting on patient preference studies
1.1 Research questions	The questions and topics that regulatory representatives hope patient preference studies will answer and the patient preference information they would find valuable to know
1.2 Sample considerations	Factors to take into account when determining whose preferences should be measured
1.3 Methodology	Considerations for the methods and instruments in patient preference studies according to regulatory representatives
1.4 Organizational considerations	Considerations for the methods and instruments in patient preference studies relating to budget, duration and timing along the medical product life cycle
1.5 Stakeholder roles in patient preference studies	Stakeholder responsibilities and collaborations in patient preference studies according to regulatory representatives
1.6 Reporting patient preference studies to evaluators	How should patient preference studies aiming to inform decision-making be described and reported to evaluators?
2. Use of patient preferences in regulatory decision-making	What do regulatory representatives think about using patient preferences in regulatory, including their value, the methods to measure them and the potential application of patient preferences in regulatory
2.1 Value of patient preferences in regulatory processes	The (added) value of patient preferences in regulatory processes and factors or situations influencing this value
2.2 Current conceptualization of patient preferences in regulatory processes	How are patient preferences currently captured in regulatory processes
2.3 Position of patient preferences in regulatory processes	How do regulatory representatives see the use of patient preferences from (conducted) patient preference studies
2.4 Remaining questions	Questions regulatory representatives raised but could not answer related to the use of patient preferences in their processes
3. Applications of patient preferences outside regulatory processes	What do regulatory representatives say about using patient preferences outside their own processes

III.IV HTA focus group themes

Themes	Definition
1. Designing, conducting and reporting patient preference studies	Topics highlighted by HTA representatives to consider in designing, conducting and reporting on patient preference studies
1.1 Research questions	The questions and topics that HTA representatives hope patient preference studies will answer and the patient preference information they would find valuable to know
1.2 Sample considerations	Factors to take into account when determining whose preferences should be measured
1.3 Methodology	Considerations for the methods and instruments in patient preference studies according to HTA representatives
1.4 Organizational considerations	Considerations for the methods and instruments in patient preference studies relating to budget, duration and timing along the medical product life cycle
1.5 Stakeholder roles in patient preference studies	Stakeholder responsibilities and collaborations in patient preference studies according to HTA representatives
1.6 Reporting patient preference studies to evaluators	How should patient preference studies aiming to inform decision-making be described and reported to evaluators?
2. Use of patient preferences in HTA	What do HTA representatives think about using patient preferences in HTA, including their value, the methods to measure them and the potential application of patient preferences in HTA
2.1 Value of patient preferences in HTA	The (added) value of patient preferences in HTA and factors or situations influencing this value
2.2 Current conceptualization of patient preferences in HTA	How are patient preferences currently captured in HTA
2.3 Position of patient preferences in HTA	How do HTA representatives see the use of patient preferences from (conducted) patient preference studies
2.4 Remaining questions	Questions HTA representatives raised but could not answer related to the use of patient preferences in their processes
3. Applications of patient preferences outside HTA	What do HTA representatives say about using patient preferences outside their own processes

SUPPLEMENTARY MATERIAL IV – ETHICS APPROVAL

Approval was gained from the following ethics committees (registration number, country):

- Medical Ethics Committee of UZ KU Leuven/Research (S59790, Belgium)
- Commission Nationale de l'Informatique et des Libertés (2036344, CNIL, France)
- Ethik-Kommission der Friedrich-Alexander Universität (92_17 B, Germany)
- Comitato Etico Instituto Europeo di Oncologia (R587/17-IEO 609, Italy)
- Medisch Ethische Toetsings Commissie Erasmus Medical Centre (WT/ss/METC306661, The Netherlands)
- Comisia de Bioetica a Medicamentului si a Dispozitivelor Medicale (CNBMDM) (5 SNI, Romania)
- Regionala Etikprövningsnämnden Uppsala (EPN) (2017/001/1, Sweden)
- Newcastle University Ethics Committee (11307/2016, United Kingdom)
- Western Institutional Review Board (WIRB) (1-1010535-1, United States)

SUPPLEMENTARY MATERIAL V: FACILITATORS AND BARRIERS TOWARDS PARTICIPATION IN PATIENT PREFERENCE STUDIES

Patient and caregivers highlighted several factors that would influence the participation of patients in patient preference studies, these included:

- Relevance of research: LC and RA patients mentioned they are happy to participate in studies regarding useful and relevant research: *"Actually, it was questions that I did not felt had relevance. A questionnaire regarding drug preferences I think I would be more engaged in"* (PA2_SE_RA).
- Having an impact: Patients from all disease areas underlined that they would participate in preference studies to have an impact. LC patients mentioned that they would like to participate in studies when they know if and what impact their contribution will have: *"Knowing this, what the survey is for, what its purpose is, otherwise it is demotivating"* (PA1_IT_LC). Different levels of impact were mentioned as incentives for participating. Patients from all disease areas mentioned the impact on their own health as an important incentive for participating: *"To be honest mine is a selfish opinion because if new things come up I hope I'll have the possibility to try them"* (PA6_LC_IT). Besides for their own health, having an impact on others' health including their own families' health, was another important incentive mentioned by participants across disease areas: *"My motivation is my youngest grandson, his prognosis is, you know, if he lasts to 20 then he is lucky"* (PA1_DM_UK). Doing something useful and helping research overall was mentioned as another important incentive and more specifically an impact on: quality and prices of medical products (CVD) and medical product development overall, e.g. new treatments (DM, RA) and new packages (RA).
- Motivation, interest and hope: While LC and CVD participants mentioned they would participate out of interest: "*to become aware of the types of treatment available, to acquire knowledge, information"* (PA5_IT_LC), DM participants specifically spoke about hope for improvement as a major driver for them to participate: *"You can dress it up, but it is all about hope"* (CA2_UK_DM). They also confirmed the opposite, namely that some DM patients lack interest and motivation and therefore would not participate: *"This really lights my fire and I am keen to do anything but then you've got people like my daughter, it doesn't matter what you say to her (...) you could say, 'There is a thousand pounds if you come along' and that wouldn't motivate her at all"* (PA1_UK_DM) (see also below: financial compensation).
- Encouragement of caregivers: LC and DM participants underlined how important the support and encouragement of family is to stimulate patients to participate in studies. DM participants in particular, stressed that often patients need to be motivated or even pushed by a family members to attend meetings and studies of any kind: *"I am only here because of my carer and wife, [name of wife], who hasn't got DM1 [Myotonic dystrophy type 1] and she said, 'You are going to this thing' "* (PA1_DM_UK).
- Relationship with recruiting person: LC and RA patients explained that the level of trust and relation they have with the person that asks them to participate influences their willingness to participate. More specifically, receiving this question from their treating physician, whom they trust, motivates them to participate: *"To encourage participation I believe also patients' trust in their oncologist is important, because when they phoned me I trusted them so I was willing to participate."* (PA5_LC_IT).
- Self-perception and disease acceptance: According to DM participants however, young DM patients are often self-conscious about their illness and do not want to attend big meetings where severely affected DM patients are present, as it confronts them with their disease: *"Young people, sort of teen onwards, they are very self-conscious. They are very aware that they have got an issue and with my two, they don't like discussing it and don't want to be in an environment with other people, particularly wheelchairs and things, it is just a turn off for them"* (CA1_UK_DM). Similarly, some LC participants mentioned that a lack of disease acceptance might hinder participation: *"There are people who hide their disease, they receive the treatment but don't say it"* (PA7_IT_LC).

- Interaction with patients: LC and DM participants mentioned that they are motivated to participate because of the possibility of sharing information, comparing experiences and just to go out and meet people: *"So if I have to say why I would encourage someone else to attend, it is because you meet other patients and you realize you are not alone"* (PA3_IT_LC). However, this view was not supported by some DM participants, who argued that DM patients often do not want to attend big meetings (see below: self-perception and disease acceptance).
- Financial compensation: Diverging views were expressed about whether or not financial compensations would incentivize patients to participate in preference studies; while some CVD participants argued that financial compensations are not needed, because they have a personal interest and "gain" from participating in preference studies, and because of the convenience of participating in preference studies situated in hospitals: "*But this is not something you should get paid for, because you don't make any effort"* (PA2_RO_CVD*),* one RA participant argued that financial compensations should be provided if the preference study interferes with work and daily activities: *"I think that if people are paid, because everyone is working and is busy and... I think that it is something like this, that is, that one gets a benefit from it personally"* (PA1_SE_RA).
- Convenience: Participants from all disease areas mentioned different aspects impacting their participation in preference studies related to the convenience of the study. According to RA and DM patients, the time investment of participation should be kept at a minimal level and hence, questionnaires should be as short as possible. One RA patient added that longer questionnaires, interfering with work life, should be financially compensated. Physical constraints (such as sleepiness and digestive problems), a long travel distance and an inconvenient time during the day were barriers to participating mentioned by DM patients: *"It is a physical constraint and that is not got to do with her willingness or not to participate, it is just the physical side, this continuing fatigue"* (PA1_UK_DM). Some DM, CVD and LC patients suggested organizing preference studies in a hospital setting, as this would allow them to combine their participation with hospital stays or visits: *"We did not give up a profession, a meal, a study, an activity of sorts to come here, we stay with you for 7 days or 10 days, for as long as we are hospitalised"* (PA2_RO_CVD). Some DM participants added that besides hospitals being *"the easiest place for them to go to"* (CA1_UK_DM), they are also familiar to DM patients.
- Attractiveness: DM participants spoke about how some young DM patients dislike big events where they are confronted with their illness. In order to increase the attractiveness and hence, the participation of young DM patients into the preference study, some DM participants suggested de-formalizing the preference study: *"If you could de-formalise it (...) something like a barbeque or a party (...) music, they all love music"* (CA2_UK_DM).
- Feedback on results: DM participants argued that receiving information on the study results would make it worthwhile for them to participate: *"It would be nice to know, get some global feedback and individual feedback because it comes back to this apathy that for me, you start to think, 'Oh, it is not worth it'"* (PA1_UK_DM). Further, this information ideally comes in the form of a short summary: *"I don't want a massive amount. A few short summaries is what I would be looking for rather than the detail"* (CA2_DM_UK).
